# Age related decline in female lar gibbon great call performance suggests that call features correlate with physical condition

**DOI:** 10.1186/s12862-015-0578-8

**Published:** 2016-01-05

**Authors:** Thomas A. Terleph, S. Malaivijitnond, U. H. Reichard

**Affiliations:** Department of Biology, Sacred Heart University, 5151 Park Ave, Fairfield, CT 06825 USA; National Primate Research Center of Thailand, Saraburi, Thailand; Department of Biology, Faculty of Science, Chulalongkorn University, Bangkok, Thailand; Department of Anthropology and Center for Ecology, Southern Illinois University Carbondale, Carbondale, Il USA

**Keywords:** Primate vocalizations, Hylobatidae, Ape communication, Great call, Honest signaling

## Abstract

**Background:**

White-handed gibbons (*Hylobates lar*) are small Asian apes known for living in stable territories and producing loud, elaborate vocalizations (songs), often in well-coordinated male/female duets. The female great call, the most conspicuous phrase of the repertoire, has been hypothesized to function in intra-sexual territorial defense. We therefore predicted that characteristics of the great call would correlate with a caller’s physical condition, and thus might honestly reflect resource holding potential (RHP). Because measurement of RHP is virtually impossible for wild animals, we used age as a proxy, hypothesizing that great call climaxes are difficult to produce and maintain over time, and that older adults will therefore perform lower quality great calls than young adults. To test this we analyzed the great call climaxes of 15 wild lar gibbon females at Khao Yai National Park, Thailand and 2 captive females at Leo Conservation Center, Greenwich, CT.

**Results:**

Findings show that call climaxes correlate with female age, as young animals (*n* = 8, mean age: 12.9 years) produced climaxes with a higher frequency range (delta F0), maximum F0 frequency and duty cycle than old animals (*n* = 9, mean age: 29.6 years). A permuted discriminant function analysis also correctly classified calls by age group. During long song bouts the maximum F0 frequency of great call climaxes’ also decreased. Additional data support the hypothesis that short *high notes*, associated with rapid inhalation as an individual catches its breath, reflect increased caller effort. Older females produced more *high notes* than younger females, but the difference only approached statistical significance, suggesting that calling effort may be similar across different ages. Finally, for the first time in this species, we measured peak intensity of calls in captive females. They were capable of producing climaxes in excess of 100 dB at close range (2.7 m).

**Conclusions:**

Age and within-bout differences in the lar gibbon great call climax suggest that call features correlate with physical condition and thus the call may have evolved as an honest signal in the context of intra-sexual territorial defense and possibly also in male mate choice via sexual selection, although further testing of these hypotheses is necessary.

## Background

Territorial vocalizations may honestly indicate an individual’s resource holding potential (RHP) if call qualities are determined by physiological constraints [1, 2]. Characteristics of vocalizations, including both spectral and temporal parameters, can vary with body size, age, rank, hormonal state or fatigue level [3–13], all of which are measures of physical condition and can thus be assumed to also be correlates of RHP. Associations between physical condition and vocal qualities have been studied most often in male animals (exceptions: [14, 15]), a sampling bias that may be due to the fact that males show greater variation in RHP, because they are usually thought to be under greater sexual selection pressure than females. More direct physiological measures of RHP, such as energetic output during contests, including ventilation rate or overall O_2_ consumption (review: [16]) would be extremely useful, as they could be directly related to field-based data, such as long term territory tenure or reproductive success.

Because direct measures of RHP are difficult to obtain from wild animals under natural conditions [17], field researchers may have to rely on indirect measures, like body size, to assess physical condition. Unfortunately, body size can likewise be difficult to measure directly in wild animals, and because of this impediment, in primate studies age has been used as a proxy for body condition [6, 18]. In fact, age may be just as reliable an indicator of RHP as size, because regardless of one’s size, physical decline that influences RHP comes with advanced age. Primate respiratory function, for example, is associated with overall health [19] and progressively decreases with age [20–22]. An individual’s age should affect not only RHP but also the individual’s ability to produce physiologically demanding vocal signals, because changes in respiratory functioning associated with age include a reduction in the lungs’ vital capacity due to loss of elasticity and distensibility, and a reduction in abdominal muscle mass [23].

Measurable characteristics of song quality that are potentially influenced by lung capacity include call intensity, call rate, and note lengths [24]. These vocal qualities are predicted to change with age and can therefore serve as indirect measures of RHP. While measuring call intensity from wild animals is difficult, measuring its spectral correlates is not. Two vocal parameters associated with loud calls that may signal RHP are the maximum fundamental frequency (F0) and the degree of change in F0 within a call note (delta F0). This is because F0 is dependent on subglottal pressure [25, 26]; so frequency and intensity are not controlled independently in the loud vocalizations of mammals [26], and receivers from a variety of taxa have been shown to respond to high intensity sounds as a fitness cue [27–30]. Furthermore, in the red deer (*Cervus elaphus*), for example, females prefer male calls with a higher F0 [31], and in some primates high-ranking individuals produce loud calls with a higher F0 than lower ranking animals [10, 32]. High F0 can also signal hormonal condition: male lar gibbons (*Hylobates lar*) show a positive association between androgen levels and F0 [33], and in other primates, e.g. male chacma baboons (*Papio ursinus*), androgen levels strongly correlate with social rank, and decline following a lowering in rank [34].

In addition to loud, high F0 signals potentially indicating that an animal has large or powerful lungs, in animals with large larynxes, like most primates, reaching a high F0 also requires extreme muscular strength to stiffen the vocal folds. Thus receivers may additionally interpret loud, high pitched calls as a fitness signal associated with larynx strength, which may correspond with overall body strength [25], although to our knowledge this hypothesis has so far not been tested in primates.

Temporal aspects of loud calls can also be associated with caller condition or state. Examples include young adults producing longer calls than more senior individuals [33], and high-ranking males generating not only longer notes [10], but also higher call rates, longer bouts and greater participation in competitive calling bouts [35]. Note duration or rate may also correlate with a signaler’s immediate energy levels, potentially providing a more useful cue to an animal’s current physical condition than static body size cues [4, 36].

Gibbons (family Hylobatidae) are small Asian apes that produce loud, complex vocalizations in song bouts, and females produce the great call, often in the context of well-coordinated duets with a mate [37–39]. Great calls likely evolved for distance communication, as they are audible up to 1 km or further through the forest [40–42], although the intensity of these calls has previously been measured in only one gibbon species, the siamang (*Symphalangus syndactylus*) [43]. The great call is thought to serve primarily as a spacing mechanism between females in inter-group communication, excluding females from each other’s territories [40, 44–47], and as such has had the potential to evolve into an honest signal of RHP.

The great calls of all gibbon species have a conspicuous climax near the end, characterized by notes produced at the highest speed and/or pitch, and a peak in amplitude [48]. For lar gibbons (*Hylobates lar*) the notes of the call climax contribute substantially to inter-individual variability and span a wide frequency range [49]. The closely spaced notes of the call climax are also the loudest notes produced by females, suggesting that the climax may be difficult to produce. If this is the case, then the great call climax potentially serves as an honest signal of a caller’s physical condition and thus may have evolved in the context of resource competition between females, the social context in which great calls are often given [41], or in the context of mate choice by males.

Although age has not been verified experimentally to correlate with RHP in white-handed gibbons, in order to provide support for the hypothesis that the lar gibbon’s great call climax is an honest index of caller condition, we used age as the most appropriate and available proxy for RHP, given that physical strength generally declines with age, including a reduction in respiratory function [20–23]. We predicted that, because F0 correlates with call intensity, older animals would show (i) a lower maximum F0 frequency, (ii) a lower F0 frequency range in the highest pitched climax note (delta F0), and (iii) a smaller ratio of note to inter-note interval durations (duty cycle). If these climax parameters are constrained by a caller’s condition and thus RHP, it should become increasingly more difficult for an aging individual to maximize them, relative to a younger individual. We also predict that, if older animals have more difficulty maintaining robust call climaxes, (iv) this will be revealed by an increase in brief, high-pitched inspirations, hereafter referred to (in italics) as ‘*high notes’*. Our previous research has shown that *high notes* occur between the longer, regular expiration notes of lar gibbon great calls, in particular following the climax notes that rise in frequency or that are maintained at high frequency and intensity [49]. They result from brief inhalations as the animal “catches its breath” between longer notes, with an average of two *high notes* occurring per great call (range: 0–5) [49]. We tested the hypothesis that *high notes* correspond with an increased vocal effort by analyzing the length and F0 frequency of great call notes that immediately precede *high notes*, versus great call notes that do not, predicting that (v) *high notes* will follow longer, higher pitched great call climax notes that span a wider frequency range.

To further test the hypothesis that our measures of the great call climax are potential indices of RHP, we analyzed how vocal parameters change within individual singing bouts, predicting that (vi) as a bout of great calls progresses, the maximum F0, delta F0, and climax duty cycle will decrease, and the number of *high notes* will increase, possibly because an individual’s vocal ability becomes taxed over the course of singing.

Finally, we also report, for the first time in this species, the peak intensity of great call climaxes, using measurements from captive animals. By quantifying the high intensity of great call climaxes, we lend support to the hypothesis that production of great call climaxes pushes females to their physical limits.

## Methods

### Subjects and recordings

We made recordings from a large group of adult female lar gibbons, spanning a wide age range (9–45.2 years). 15 of the subjects were wild animals, recorded at the Mo Singto—Klong E-Tau long-term research site at Khao Yai National Park, Thailand (101°22’E, 14°26’N), 130 km NE of Bangkok in June-July 2013 and July-August 2014, under a research permit issued by the National Research Council of Thailand (NRCT) and the Department of National Parks, Wildlife and Plant Conservation (DNP) of Thailand. All wild lar gibbons have been habituated to the presence of researchers for decades, in some cases for their entire lifetime. We also obtained recordings from two captive female lar gibbons at the LEO Zoological Conservation Center in Greenwich, Connecticut, USA. Each captive animal was housed with an adult male in one of two large outdoor enclosures. We made all recordings with a digital solid-state recorder (Marantz PMD661, Kanagawa, Japan) and a Sennheiser microphone: K6 power module and ME67 recording head and windscreen (Sennheiser Electronic, Wedemark, Germany). All recordings were 24 bit, with a 48 kHz sample rate.

All research was approved by the Institutional Animal Care Committee of Sacred Heart University, and adhered to the American Society of Primatologists Principles for the Ethical Treatment of Non-Human Primates. We avoided direct interaction with animals in all cases, but to minimize recording distances we usually approached wild gibbons close enough to record from directly underneath the tree in which an individual was vocalizing (<50 m), and in direct line of sight. We recorded all calls between 6:00 and 11:00 AM, as the females engaged in morning duets with males, often in response to the calls of nearby animals. We identified individual wild animals by territory location and individual pelage color and markings based on long-term photo-records and long-term knowledge of individuals [50].

### Age estimates

We determined the ages of the two captive animals from birth records. The wild gibbon population has been studied for decades and all females included in our analyses have been monitored continuously since they were first recorded in the study population [50–53]. Precise birth records were available for six females within a window of accuracy of ±53.8 days (range 2–120 days; Table 1). For the other wild females (*N* = 9) we estimated a minimum age, based on reproductive history. One female (R) was first seen as a subadult in her natal group and her birth was back-dated based on her estimated age. For the remaining 8 females, minimum age was calculated based on long-term records of reproductive history. We used age at first reproduction as the most objective measure to calculate a female’s minimum age because, compared to other developmental landmarks, age at first reproduction has shown low inter-individual variation in our population [53]. Four females with unknown birth dates (A, H, NOS, S) were residents when long-term monitoring of the population began. For these females, we used date of first observation and reproductive history to calculate minimum female age. We assumed that each female was the mother of all non-adults in each group at the time of first observation and that the oldest non-adult was always the female’s first offspring. We then subtracted the age of the oldest non-adult in the group from the date the female was first recorded. For example, adult female NOS was first observed in September 1999 when she was carrying a 1-year old infant, which was the only non-adult in the group. Subtracting 1 year from September 1999 resulted in back-dating the offspring’s birth to September 1998. To estimate the female’s minimum age, we further assumed that females in our population do not give birth before the age of 10.5 years, which is the average age at first reproduction, based on long-term demographic records [53]. Subtracting 10.5 years from the female’s assumed date of first reproduction then gave a minimum birth estimate for the female herself. In the case of adult female NOS, subtracting 10.5 years from September 1998 indicated that the female was born not later than March 1988. Thus, when we recorded her song in June 2013 she was at least 25 years 3 months old (Table 1). For a second group of young females (*N* = 4), for whom we could not know birth dates (BD, C, D, E) because they immigrated into the study population as adults, we likewise based age estimates on first reproduction by subtracting 10.5 years from the time of these females’ recorded first parturition (Table 1).

**Table 1:** Ages of all females studied

Female	Age at first vocal recording (years/months)	Window of accuracy (days)
**Females with known birth date (** ***N*** **= 8)**		
**T**	**27/6**	**±120**
**So** ^**a**^	**25/9**	-
**G**	**23/7**	**±90**
**N**	**17/7**	**±60**
*M*	*16/8*	*±2*
*J*	*15/0*	*±30*
*W*	*11/5*	*±21*
*Te* ^*a*^	*9/0*	*-*
**Females with estimated birth date (** ***N*** **= 9)**		
**A**	**45/2**	-
**S**	**38/2**	-
**H**	**34/3**	-
**R**	**29/5**	-
**NOS**	**25/3**	-
*D*	*16/3*	*-*
*E*	*13/5*	*-*
*C*	*11/7*	*-*
*BD*	*10/2*	*-*

^a^denotes a captive female. The names and ages of young females (< the median Age of 17 years and 7 months) are indicated by italics. The names and ages of old females are in bold

Our method of calculating female age based on first parturition potentially underestimates the age of these 4 animals, if individuals had a birth prior to their natal dispersal and/or before their emigration into the study population. However, secondary female dispersal has not yet been observed in the Khao Yai white-handed gibbon population and our approach to calculate female ages was conservative as it resulted in minimum ages. Moreover, a margin of error of only a few years is unlikely to be consequential, particularly for the purpose of grouping animals into the broad categories of young and old, given the long lifespan of the species and the broad range of ages sampled. The maximum lifespan of captive Hylobates has been reported to be 35.6 years [54], but this is an underestimate, as two of the wild subjects in our study exceeded that age, at 38.2 and 45.2 years, and a captive gibbon (*H. muelleri*) has been reported to have lived 60 years [55].

### Measurement parameters

**Fig. 1:**
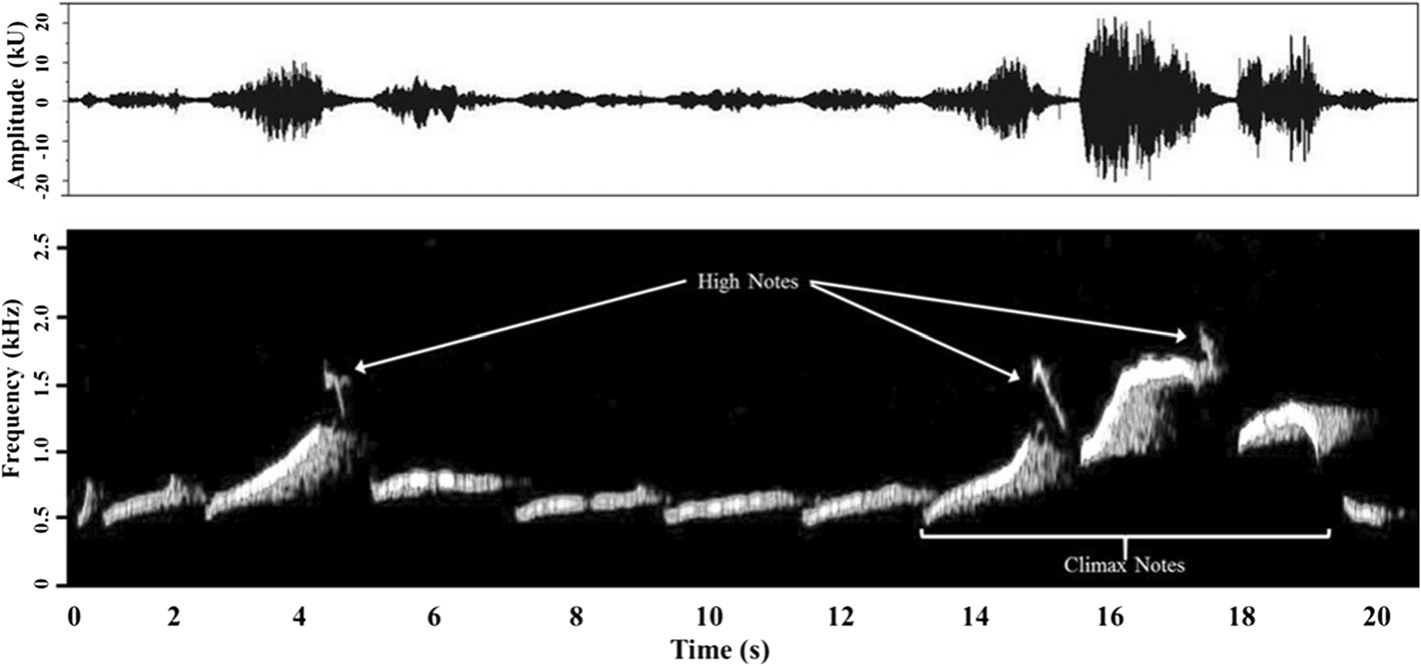
Waveform (top) and spectrogram (bottom) of the lar great call, from a 24 year old female. Three *high notes* are indicated, and represent the sounds associated with inhalations between great call notes. The three notes of the climax are also labeled. Window type: Hann, FFT size: 1124 Hz, frame overlap: 50 %. Harmonics and background noise have been removed from this image

We converted all recordings of great call climaxes into spectrograms (Window type: Hann, FFT size: 512 hz, frame overlap: 50 %), using Raven Pro 1.5 Sound Analysis Software (Cornell Lab of Ornithology Bioacoustics Research Program, Ithaca, New York), and made measurements from the spectrograms. The amplitude and frequency of the lar gibbon’s great call climax rises and falls over multiple notes [42, 49]; we defined it as consisting of three notes: the expiration note that reaches the highest frequency of the great call, and the notes that immediately precede and follow it (Fig. 1). We measured frequency and temporal parameters from 230 great calls (mean: 13.5 calls per animal; range: 2–27). The frequency measures were maximum and range (delta) of F0 frequency for the highest note of each climax, as well as the lowest F0 frequency of the great call climax. The temporal measure was the duty cycle of each climax, and was calculated as the total duration of the three notes in a climax (signal) divided by the signal plus the sum of the inter-note intervals that followed each climax note. A larger duty cycle thus represents longer climax note output relative to the breaks between notes. We also compared the total number of *high notes* (rapid inspirations) per call, across all animals.

In addition, we combined the measures described above to create an index of call quality for each animal, to obtain a single measure that could be compared across all ages. We created the index by ranking animals, from 1 to 17 (*n* = 17 animals), in each of the first three measurement parameters (delta F0, maximum F0 and duty cycle). The lowest rank for a given measure, a rank of 1, was thus assigned to the animal that had the smallest value of that measure, and a rank of 17 (or the highest rank) to the animal with the highest value. Consistent with our predictions, *high note* production was ranked in the reverse order, so that the lowest ranked animal produced the greatest average number of *high notes* per call. All four ranking positions were then averaged for each animal, to produce its individual ranking index.

Two age groups were compared, using separate between-subject t-tests (two tails), to test the hypothesis that broadly differing age classes are distinguishable, even if more precise ages cannot always be predicted based on call characteristics. Too few subjects were available to divide the sample further. Thus, all animals below the median age of 17.6 years were categorized as young adults (*n* = 8, mean age: 12.9 years, range: 9–16.7 years), and all others as old adults (*n* = 9, mean age: 29.6 years, range: 17.6–45.2 years) (Table 1). For each of the measures we first calculated within-animal means, and then compared these means across all 17 animals. We corrected for multiple t-tests using the method described by Benjamini and Hochberg [56], which adjusted the P level of significance from 0.05 to 0.0375. In order to explore the overall relationships between age and the call parameters across the entire population, we also made separate regressions for each measurement parameter, as a function of caller age.

In order to determine how often great calls can be accurately assigned to either the young adult or old adult age groups, we also used the call parameters in a nested, permuted discriminant function analysis (pDFA) [57]. We conducted the pDFA using a function written in R [58] by R. Mundry, and based on the function Ida of the R package MASS [59]. Delta F0 was omitted as a classification parameter for this analysis, as it was highly correlated with the maximum F0 frequency measure. Three animals (D, E and G) were also excluded from the analysis because their call sample sizes were too small (2–3 calls). The remaining 14 animals had a mean of 15.9 ± 1.8 great calls per animal (range: 6–27).

In order to test the hypothesis that *high notes* correspond with an increase in vocal effort, we used separate paired T-tests to compare the delta F0, maximum F0, and the note duration of climax notes, depending on whether or not they were followed by a *high note*. We predicted that when a female exaggerates a climax note by increasing these parameters, it will require her to produce a rapid inspiration (*high note*), and thus *high notes* will follow longer notes that span a wider frequency range and reach a higher pitch.

The results of our comparison of song parameters by age allowed us to predict that these parameters would change over the course of song bouts, within individuals. We were able to record bouts containing ≥ 8 great calls from seven females, all from the wild population (mean: 9.9 calls per bout; range: 8–12, mean bout length 32.8 min, range 12–54 min). Two animals produced two bouts each; for each of these animals, we averaged the note parameters from each of their bouts and used those averages to represent that animal in subsequent comparisons. We calculated the mean delta F0, maximum F0, climax duty cycle and number of *high notes* per great call from each animal’s bout. Since bouts differed in the number of great calls produced, in order to generate regressions that compared measurement parameters over the course of bouts, we plotted each parameter over the last 7 great calls of a given bout, as a percentage of the average value obtained from the calls that preceded these 7 calls, at the start of the bout (*n* = 1–5 great calls, depending on the number of calls in a given bout). We also compared parameters obtained from the first four and last four great calls of bouts, predicting that delta F0, maximum F0, and duty cycle would be decreased, and *high note* production increased from early to late in bouts. We used matched-sample, two-tailed t-tests to compare the early versus late bout parameters.

### Intensity measures

We recorded the peak intensity (dB SPL, A scale, 125 ms ‘fast’ time weighting) of great call climaxes from the two captive animals (ages 9.0 and 25.9 years) in their outdoor enclosures, each housed with an adult male. We obtained dB measures with an SPL meter (Checkmate CM-150, Galaxy Audio, Wichita, KS) with a built in ½ inch electret condenser microphone and a windscreen (frequency range 31.5Hz-8KHz, measuring level range 30-130 dB, accuracy ±1.5 dB), and only collected data when the animals were oriented towards the microphone. Animals were separated from the microphone by wire mesh, all calls were produced and recorded from heights of over 2 m, and no major sound reflective structures were in the vicinity of the recording site, so the data are essentially free-field. As subjects were free to move within their enclosures, we had to measure from a range of distances, between 2.7 and 6.1 m. In order to control for amplitude differences due to the effect of spherical spreading, the dB of all calls obtained from distances greater than the nearest (2.7 m) call were adjusted to that distance standard by applying the inverse distance law. In order to calculate the mean dB of all peak call notes for a given individual, each dB measure was converted to intensity, these intensity measures were then averaged, and that average was then converted back into an average dB measure. Peak call amplitudes were compared between the two animals with a two-tailed between-subjects *t*-test.

## Results

### Age and great call climaxes

**Fig. 2:**
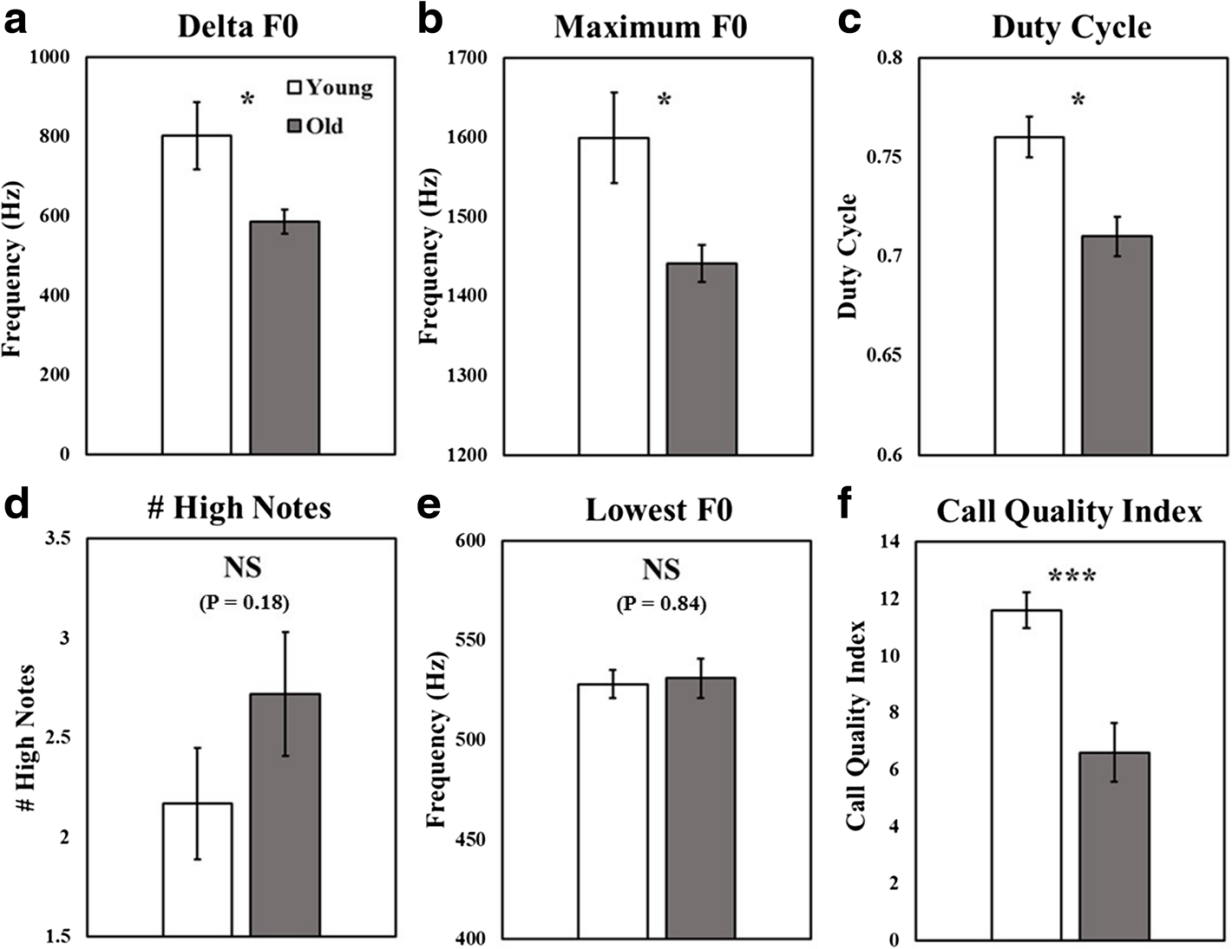
Comparisons of great call parameters between young adults (white bars) and old adults (gray bars). The overall call quality index (**f**) summarizes rankings derived from four parameters: delta F0 (**a**), maximum F0 (**b**), duty cycle (**c**) and number of *high notes* (**d**). Error bars represent ± 1 SEM. *P < 0.05, ***P < 0.001, NS: not significant

The differences in call parameters between the two age groups, which we describe in this section, are summarized in Fig. 2. Young adult females had a larger delta F0 (mean 802 ± 84 Hz, range 569 – 1206) than old females (mean 586 ± 31 Hz, range 416 – 692, T (15) = 2.70, *p* = 0.031). Similarly, a significant inverse relationship (*r* = −0.484) existed between delta F0 of the peak climax note and caller age (F (1, 15) = 4.58; *p* = 0.049). Young adults also produced significantly higher maximum F0 frequencies (mean 1599 ± 57 Hz, range 1387 – 1788) than old adults (mean 1441 ± 23 Hz, range 1329 – 1546, T (15) = 2.88, *p* = 0.02), but an inverse relationship (*r* = −0.474) between the maximum F0 of great call climaxes and caller age barely failed to reach statistical significance (F (1, 15) = 3.99; *p* = 0.06).

The maximum F0 effects described above may be due to a general lowering of voice in older females, rather than solely due to a failure to reach higher pitch in the peak notes. Lowering of voice has been identified in other female primates, as a correlate of increased body size [60], but it is not known if gibbons increase in size throughout adulthood. In order to investigate the possibility that overall F0 decreased with age, we also measured the lowest F0 frequency of the climax, but found no difference between the age groups. The lowest F0 frequency was 528 ± 7 Hz for the young adult group and 531 ± 10 Hz for the older females (T (15) = 0.20, *p* = 0.84). Similarly, there was no correlation between this measure and caller age, across the entire population (*r* = 0.006) (F (1, 15) = 0.001; *p* = 0.98). Based on this result, it is unlikely that the lower maximum F0 of old females was related to a general lowering of voice with increasing age, but rather to a failure to produce high climaxes.

Great call climaxes from young adult females had a higher duty cycle (mean 0.76 ± 0.01, range 0.73 – 0.81) than those of old females (mean 0.71 ± 0.01, range 0.66 – 0.79) (T (15) = 2.98, *p* = 0.01), meaning that young females had a longer duration of notes relative to the intervals between them. The overall negative correlation (*r* = − 0.425) across the entire population, however, barely failed to reach significance (F (1, 15) = 3.32; *p* = 0.09).

In contrast to the F0 and duty cycle measures, where young females were expected to show higher performance, old females were predicted to produce more *high notes*. Since *high note* averages were derived from count data, we confirmed that our *high note* data were normally distributed before we analyzed them, by performing a Shapiro-Wilk W Test (W = 0.95, *p* = 0.47). Although a trend for young females to produce fewer *high notes* than old females existed, it was not significant. The mean rate of *high note* production by young adult females was 2.17 ± 0.28 and that of the old females was 2.72 ± 0.31 (T (15) = 1.38, *p* = 0.18). We also did not find a significant correlation between age and the number of *high notes* produced per great call (r = 0.36) (F (1, 15) = 2.19; *p* = 0.16).

**Fig. 3:**
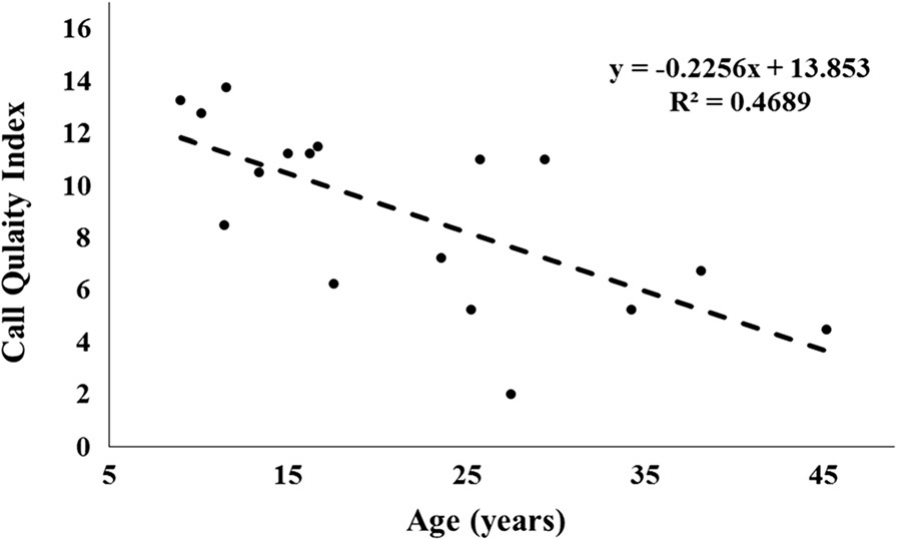
Call quality index by caller age. The call quality index is based on rankings within the population in regards to four characteristics of the female great call: The maximum F0 frequency of the climax, the delta F0 frequency of the climax, the duty cycle of the climax, and the number of *high notes* produced

The overall call quality index, a summary of the call features described above, differed between the young (mean 11.6 ± 0.63, range 8.5 – 13.75) and old females (mean 6.6 ± 1.03, range 2 – 11): T (15) = 4.25, *p* < 0.001. The call quality index rankings also showed a negative correlation (r = − 0.68) with caller age (F (1, 15) = 13.3; *p* = 0.002), Fig. 3. The permuted discriminant function analysis, used to discriminate great calls by age group, correctly classified the calls into their respective age group at a rate significantly greater than chance (62.4 %, *p* = 0.022).

### *High note* inspirations follow longer climax notes that span a wider frequency

**Fig. 4:**
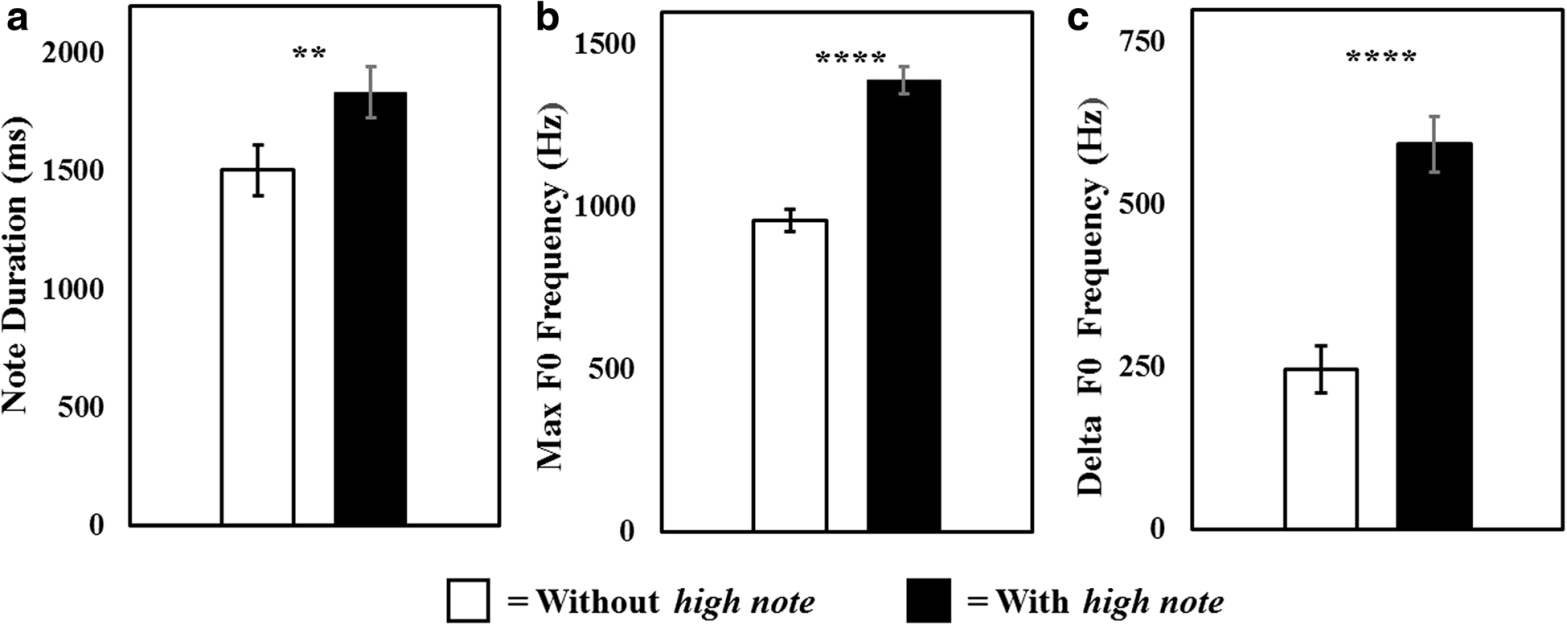
Analysis of climax notes that are followed by *high note* inhalations versus those that are not, within animals. Climax notes that are followed by *high notes* are longer (**a**), have a higher maximum F0 frequency (**b**) and a higher delta F0 frequency (**c**) than notes that are not followed by *high notes*. White bars: climax notes not followed by a *high note*, Black bars: climax notes followed by a *high note*. Error bars represent ± 1 SEM. **: *P* < 0.01, ****: *P* < 0.0001

Within individuals, climax notes that were followed by *high notes* were longer (mean: 1833 ± 108, range: 1520 – 2923 ms) than those not followed by *high notes* (mean: 1506 ± 67, range 1050 – 1819 ms) (T (16) = 3.5, *p* = 0.003) (Fig. 4a). In addition, when followed by *high notes*, climax notes had a higher maximum F0 frequency: (mean: 1388 ± 43, range: 1128 – 1757 Hz, versus: 957 ± 36, range: 749 – 1207 Hz) (T (16) = 9.7, p < 0.0001) (Fig. 4b), as well as a higher delta F0 (mean: 593 ± 47, range: 323 – 1206 Hz, versus: 247 ± 19, range: 146 – 386 Hz) (T (16) = 7.68, *p* < 0.0001) (Fig. 4c).

Because *high notes* are produced by inspiration and more often follow longer expiration climax notes of higher maximum F0 and delta F0, *high notes* may reflect a greater effort invested in producing the note that precedes them. *High notes* may be produced involuntarily, as a female rapidly inhales to refill lungs that were depleted by the preceding note. Thus, the production of *high notes* potentially indicates that a female is having difficulty producing a high quality climax, and that females with waning stamina may be forced to produce more *high notes* during a song bout.

### Climaxes change within song bouts

Maximum F0 decreased over the course of song bouts (*r* = − 0.78) (F (1, 6) = 9.3; *p* = 0.023). Mean maximum F0 at the beginning of bouts was 1488 ± 66 Hz, and at the end 1430 ± 67 Hz. (T (6) = 2.79, *p* = 0.032). This frequency decrease across bouts is consistent with the notion that the production of high frequency call climaxes may be difficult to sustain. A trend of decreasing delta F0 over the course of song bouts barely failed to reach significance, (r = −0.69) (F (1, 6) = 5.5; *p* = 0.057), and mean delta F0 did not significantly differ between the beginning (640 ± 118 Hz) and the end (566 ± 74 Hz) of bouts: (T (6) = 1.13, *p* = 0.30). A trend of increasing *high note* production from the beginning (1.63 ± 0.32) to the end of bouts barely failed to reach statistical significance (1.84 ± 0.3) (T (6) =2.12, *p* = 0.078), and the overall trend over the course of the entire bouts (r = 0.66) was also not significant (F (1, 6) = 4.7; *p* = 0.074). We did not find a significant trend in duty cycle change over the course of bouts (*r* = 0.13) (F (1, 6) = 0.11; *p* = 0.11). Duty cycle was 0.679 ± 0.04 at the beginning of bouts, and 0.683 ± 0.04 at the end (T (6) = 0.39, *p* = 0.70). Extended great call bouts thus result in a decrease in maximum F0, but not in robust changes to the other call parameters.

### Climaxes can exceed 100 dB

We also measured the intensity of great call climaxes. Each of the two captive animals from whom call intensity was measured was capable of producing climax notes in excess of 100 dB SPL at 2.7 m. The younger animal’s peak call intensity (mean = 105.3 dB, range 101.4-106.9 dB, *n* = 3) was higher than that of the older female (mean = 98.5 dB, range 80.3-103.5 dB, *n* = 11): t (12) = 2.2, *p* = 0.047), demonstrating that maximum call amplitude can differ between individuals. Whether or not such differences are associated with age will require analysis of a significantly larger sample. This finding reveals that the amplitude of lar gibbons’ great calls is comparable to vocalizations that are known for their high intensity from other species, such as the Mantled howling monkey (*Alouatta palliata*) calls: >90 dB at 5 m [61], or the roars of lions (*Panthera leo*): approximately 114 dB at 1 m [62].

## Discussion

Our study is the first to demonstrate the effect of age on the production of gibbon great calls. The great call is arguably the most significant vocalization in the female gibbon’s vocal repertoire, in part due to its long-range propagation. If the differences in the call parameters that we measured correlate with physical condition, then young females may be capable of signaling high RHP to potential rivals. These hypotheses have not yet been fully tested, but our interpretation is compatible with field observations of female-female replacement, because among the females of known ages that have been observed to date, only young females have replaced old females, and not *vice versa* (*N* = 3; [53]). Here we have analyzed cross-sectional data of vocalizations from multiple individuals, but in order to provide a more reliable estimation of long-term within-individual variation, a longitudinal study involving data collected at different ages from the same individuals would be needed. Cross-sectional studies run the risk of being biased by differential mortality [63], but that is not likely to be the case for the species we studied, which is very long-lived, with low adult mortality.

Within-bout changes to the climax of the territorial great call and measures of the high intensity of climaxes support the hypothesis that these vocalizations are produced at the limit of callers’ vocal abilities, and thus may indicate a caller’s condition or stamina. We do not yet know if this is the case, and further testing of this hypothesis would be technically challenging. Appropriate tests would require experiments that directly measure individual physical condition and relate it to our great call measures, in addition to field data showing that great calls predict, for example, territory size, quality, length of tenure on a territory, or lifetime reproductive success. Nevertheless, additional characteristics of the great call climax, and the contexts in which it occurs, are in line with the hypothesis. Climaxes have been described as “penetrating and unmusical” [40], and, unlike the other notes of the species’ repertoire, often contain multiple harmonics and rapid jumps in pitch, unstable and noisy qualities that correspond with the chaotic vocal-fold vibration that typically occurs when a mammal’s vocal folds are taxed by reaching their amplitude and/or frequency limit [64, 65]. Great calls also often fail to reach full pitch on the climax [40], climax notes are sometimes ‘left out’ while the preceding rising and following falling notes are still produced (pers. observ.), and when calls are occasionally stalled or entirely aborted, it is typically just before the climax [38], further suggesting that the climax is the most difficult part of the call to produce. It is also noteworthy that, during development, young adolescent and subadult lar females may practice great calls in temporal synchrony with their mother’s calling, yet usually do not produce the climax portion of the call until they are fully mature or, more often, until they sing independently of their mother [66, 67].

The great call differences by age that we have documented could reflect differences in caller motivation rather than physical ability, what game theoretical models describe as ‘aggressiveness signaling’ [68]. For example, older adult females may produce softer, lower-pitched climaxes to avoid conflicts with neighbors, and not because they are incapable of producing more robust calls. Alternatively, younger adults may be more motivated to call because they have more recently acquired a territory and thus may have more to lose than an older individual who has been a long-time resident. Territory ownership is critical for female reproduction, because over the approximately three decades that the population analyzed here has been monitored, no female has successfully reproduced who was not a resident on a territory. Our findings, however, suggest that it may not be the case that the age differences in call climaxes that we have found are related to motivation. *High note* inhalations likely correlate with caller effort because they follow climax notes, the loudest notes that female lar gibbons produce [49], and as we have shown here, when an individual produces a *high note*, it follows a climax note of higher average frequency, higher delta F0 and longer duration. Thus, since both young and old animals produced a similar number of *high notes* in their great call climaxes, they likely exert a similar effort to produce them. If the effort exerted in call production remains high throughout life, then the differences we have measured in other call parameters are more likely to be attributable to age-related physiological decline than to motivation. Similarly, *high notes* could potentially maintain a signal’s honesty throughout life and even within bouts, if attempts to exaggerate a climax result in the production of more of these notes, thus revealing to listeners the difficulties an individual of comparatively lower quality may have producing a climax. Playback studies would be an effective method of testing the functional significance of *high notes*.

If the great call climax contains cues that make it an honest index of a caller’s physical condition, this does not guarantee that other animals attend to such cues, or that they are even capable of detecting the differences that we describe. It is theoretically possible that evolutionary constraints have acted to enforce honest call climaxes, but in the absence of selection specifically for honesty [24]. However, as loud territorial calls that correlate with RHP have been shown to influence conspecific behavior in multiple taxa [8–10, 28–30], and because there is strong competition between gibbon females for possession of a territory [41], which was likely the ultimate reason for hylobatids living in pairs [69, 70], it is reasonable to expect that an evolutionary pressure existed for female calls to signal RHP. The current study did not test whether listeners can detect and differentially respond to the great call features that we have quantified, for which the most appropriate test would be to measure responses to great calls in playback experiments. Field observations do, however, suggest that other animals attend to the climax. The female’s mate usually ceases calling until just after the great call climax [71], and neighboring animals also often pause their own duet singing until after the conclusion of another female’s great call climax (pers. observ.). The fact that males stop their calling to listen to neighboring females’ call climaxes further suggests that one possible function of the great call may be to advertise a female’s condition to prospective mates outside of her current pair bond. This is in line with mounting evidence that lar gibbons employ a mixed reproductive strategy that includes females mating polyandrously, both within multi-male groups, with neighboring males from outside the group, and with floaters arriving from more distant locations [33, 52, 53].

Psychophysical data on primate auditory perception suggest that the maximum F0 and delta F0 differences that we quantified here (the smallest being the change in maximum F0 frequency within bouts, at approximately 4 %) are large enough to be detectable by conspecific listeners. In general, auditory thresholds are similar across many primates [72], but still await measurement for gibbons. Macaques (*Macaca fuscata* and *M. mulatta*) can detect frequency changes of approximately 2–3 % for pure-tone stimuli below 2 kHz [73, 74]. Frequency discrimination is similar for baboons [75], and even greater for humans [73]. Hence, if lar gibbons show frequency sensitivity like that of other primates, the variation in maximum F0 and delta F0 that we report here between age groups and over the course of bouts should be detectable. Similarly, detecting the production of *high notes* is likely to be trivial for immediate neighbors, as human observers easily hear *high notes* from distances that are typical of encounters between neighboring animals [49]. But *high notes* should not be discriminable at greater distances, as they are of high frequency and low amplitude and therefore attenuate rapidly in a forest environment. If *high note* detection is only possible between immediate neighbors, then those neighbors may be able to discriminate calls more accurately than distant animals, who generally pose more of a threat to one’s territory than an immediate neighbor [76]. However, if *high notes* are involuntarily produced from rapid inhalation when a female “catches her breath” after producing a robust climax note, then natural selection would likely act to minimize this potential cue of low RHP. More tests are needed to explore the significance of great call *high notes*, especially because we did not find age differences in their production.

Our measurements of peak call intensity were all obtained from the highest pitched expirations of the call climax, demonstrating that intensity and frequency are correlated in the great call, and likely interdependent. Similarly, a positive correlation between amplitude and F0 occurs in loud vocalizations from multiple taxa [10, 77, 78], and high F0 has been positively associated with social rank as well [10]. As amplitude and F0 are often correlated, receivers could theoretically attend to either one, or both parameters. For lar gibbon great calls, however, maximum F0 is likely to be more reliably transmitted to receivers than intensity. This is because call intensity should vary much more than frequency, both between and within calls, as the caller is often changing her directional orientation. These changes are in fact typical during climaxes, when a female may perform acrobatic locomotor displays that include leaping between tree branches [79]. Additional studies are needed to analyze the relationship between intensity and the spectral and temporal features of great calls. Of the up to 19 currently recognized gibbon species [80], call intensity has only been measured in one other species, the siamang (*Symphalangus syndactylus*) [43]. The maximum intensity reported for siamang calls was between 95 to 113 dB SPL, but measurements were taken at varying distances that were not reported for each call, so a direct comparison to the lar female’s peak call intensity, which we report here, is not possible.

Although many studies that analyze the temporal features of calls have measured note duration [10, 13, 81] or note rates [82, 83], we chose a more complex parameter, the duty cycle of call climaxes. We chose this as a potential correlate of caller age because selection does not seem to have favored either long note durations or enough notes to emphasize a rapid delivery rate in the lar gibbon call climax. In regard to note durations, in all gibbon species whose great calls have been analyzed, the climaxes are characterized by multiple notes of short duration, and this appears to be the ancestral condition for hylobatids [48, 79]. Furthermore, lar gibbon climax notes are high-pitched and loud, features associated with a rapid emptying of the lungs and therefore not expected to correlate with a maximization of note duration, especially because the climax is spread over multiple notes. We also did not expect note rate to differ by age or across bouts because, relative to other gibbon species [48, 79], the lar gibbon’s call climax has only a few notes that are produced at a relatively low rate. In contrast, even if selection has not specifically favored long notes or a rapid delivery rate in lar great call climaxes, a high ratio of note to inter-note interval duration (duty cycle) is potentially an index of caller condition because it provides a single metric that summarizes the duration in which multiple loud, high pitched call notes can be sustained, relative to the time needed to refill the lungs between them. It is reasonable to assume that potential indices of caller condition like this, and others [24], can include integrals of multiple features, especially in the context of a complex, multi-component vocalization such as the great call. Furthermore, if gibbons attend to multiple features of their neighbor’s calls, they may be able to assess age and thus potentially RHP with greater accuracy, as the overall call quality indices that we report here suggest.

Our within-bout results revealed a decrease in maximum F0, similar to the negative age-effect. But unlike the effect of age, no change in duty cycle was observed over the course of bouts. Overall, the within-bout changes that we observed support the notion that the production of great call climaxes with a high F0 cannot be sustained over prolonged bouts. Unfortunately, our within-bout sample contained too few callers to allow for age comparisons across extended bouts.

It is not known what specific proximate mechanisms are responsible for the age-related changes to vocal output that we report. Reproductive hormonal condition is one likely candidate. Although there is an association between androgen level and call qualities in male lar gibbons [33], the effect of hormones on female great calls has not yet been studied, and is technically challenging as hormone levels fluctuate considerably across the menstrual cycle [80]. Changes in female vocal quality are known to occur throughout life in humans, including during menstruation, pregnancy and menopause [85, 86], and estrogen deprivation associated with menopause changes the mucous membrane lining of the vocal tract which can lead to voice changes [23]. Hormonal state is also correlated with changes to the voice in yellow baboons (*Papio cynocephalus cynocephalus*) [87], and Barbary macaques (*M. sylvanus*) [14].

Changes to the female voice, especially those mediated by sex steroid hormones, may influence mate choice by males. Barbary macaque males, for example, respond more strongly to female calls that signal an increased probability of conception [14], and in humans the female voice is rated as more attractive by men as the probability of conception increases [88]. Similar hormonally-influenced vocal cues may be of particular importance in gibbon signaling, as they have the potential to provide information to neighboring males when visual cues, such as sexual swellings, are difficult to assess. Lar gibbons produce only moderate sexual swellings [84], and inhabit dense forest environments that limit the effectiveness of visual signals. In contrast to visual signals, the great call climax is an effective long-range signal. Although white-handed gibbons are primarily pair-living, long-term demographic data from Khao Yai show that females change a social partner several times over the reproductive life-span [53]. Thus, selection may have promoted a long-range signal in lar gibbon females that could not only signal her RHP, but also might increase her attractiveness to males in the area, thereby increasing her own reproductive success.

## Conclusion

Here we have shown that a vocal signal, the great call climax of female lar gibbons, often exceeds 100 dB SPL at close range and shows declines in frequency with age and within bouts, as well as age-related declines in temporal characteristics. If the measures that we used are predictive of an animal’s RHP, which is not yet known, then these findings suggest that the great call might serve as an index of caller condition in the context of female-female competition in territorial defense, and potentially also for attracting a mate(s). Further studies are needed to explore these possibilities.
